# Prevalence and predictors of different patterns of hypertension among adults aged 20–60 years in rural communities of Southeast Nigeria: a cross-sectional study

**DOI:** 10.1186/s13690-021-00724-y

**Published:** 2021-11-25

**Authors:** Rufina N. B. Ayogu, Mmesoma G. Ezeh, Adaobi M. Okafor

**Affiliations:** Department of Nutrition and Dietetics, University of Nigeria, Nsukka, Enugu, Enugu State Nigeria

**Keywords:** Isolated systolic hypertension, Isolated diastolic hypertension, Combined systolic and diastolic hypertension, Predictors, Adults, Rural Nigeria

## Abstract

**Background:**

Hypertension, a major cardiovascular disease risk factor exists several years without symptoms. Few data exist on prevalence and predictors of hypertension among apparently healthy Nigerian adults. This makes it difficult for policy-makers to concentrate efforts to control emerging health burden of the disease. This study assessed prevalence and predictors of isolated systolic hypertension (ISH), isolated diastolic hypertension (IDH) and combined systolic and diastolic hypertension (CSDH).

**Methods:**

Cross-sectional survey design was employed in the study of 517 adult participants (20–60 years) in a rural setting. Selection of the respondents was through multistage sampling which involved systematic, proportionate and simple random sampling. Data on socio-demographic characteristics, blood pressure, height, weight, and waist circumference were collected. Frequencies, T-test, analysis of variance and Chi square were used in statistical analysis. Bivariate and multivariate logistic regressions were used to evaluate variables associated with different patterns of hypertension with significance accepted at *P* < 0.05. Frequencies, percentages, crude and adjusted odd ratios were reported. Statistical Product and Service Solutions version 21.0 was used in statistical analysis.

**Results:**

ISH (10.6%), IDH (18.2%) and CSDH (37.8%) were observed among the participants. ISH was less likely among 20–29 year-olds (adjusted odds ratio (aOR) = 0.35, 95% confidence interval (C.I.) = 0.13–0.94), 30–39 year-olds (aOR = 0.30, 95% C.I. = 0.11–0.82) and those with abdominal obesity (aOR = 0.12, 95% C.I. = 0.03–0.56). Participants who perceived their health status as good (aOR = 3.80, 95% C.I. = 1.29–11.18) and excellent (aOR = 5.28, 95% C.I. = 1.54–18.07) were respectively 3.80 and 5.28 times more likely to have ISH. Those with secondary education had significantly higher likelihood for IDH (aOR = 2.05, 95% = 1.02–4.14) whereas self-perceived poor health status (aOR = 0.24, 95% C.I. = 0.09–0.65), absence of obesity (aOR = 0.10, 95% C.I. = 0.01–0.81) and general obesity (aOR = 0.35, 95% C.I. = 0.17–0.72) were associated with reduced risk for IDH. Secondary (aOR = 0.60, 95% C.I. = 0.36–0.99) and tertiary (aOR = 0.49, 95% C.I. = 0.28–0.85) education were associated with reduced risk for CSDH but combined obesity (aOR = 4.39, 95% C.I. = 2.25–8.58) increased the risk for CSDH by 4.

**Conclusion:**

ISH, IDH and CSDH were problems among the adults with age, obesity, self-perception of good/excellent health status and low education level as significant predictors. Health and nutrition education to prevent comorbidities and cerebrovascular accidents are recommended.

## Background

Hypertension is a serious global long term medical problem that spans across all stages of life with high affinity for adults due to cumulative effects of most risk factors. Defined as systolic blood pressure of ≥130 mmHg and or diastolic blood pressure of ≥80 mmHg [1], hypertension has been described as more serious than other risk factors of cardiovascular diseases (CVDs) partly because it is symptomless. Hypertension is a major cause of premature death worldwide due to its numerous comorbidities and associated risk of damage to vital body organs like the brain, heart and kidneys.

Systolic hypertension has been identified as more prevalent than diastolic hypertension in older adults [2] implying that diastolic hypertension may be preponderant in younger adults. Raised systolic blood pressure (SBP), a stronger determinant of cardiac target organ damage than raised diastolic blood pressure (DBP) [3] has a greater effect on angina, myocardial infarction, and peripheral arterial disease, whereas raised diastolic blood pressure (DBP) had greater effect on abdominal aortic aneurysm than raised SBP [4]. Researchers have reported higher incidences of coronary heart disease, stroke, heart failure, and peripheral arterial disease among men with isolated systolic hypertension (ISH) than those with isolated diastolic hypertension (IDH) while those with combined systolic and diastolic hypertension (CSDH) had marginally higher risk than those with ISH [5, 6]. This shows that CSDH carries the greatest risk of target organ damage followed closely by ISH and lastly by IDH.

In a review analysis of worldwide data to ascertain global burden of hypertension [7], a total of 972 million (26.4%) people were reportedly living with hypertension globally with a danger of increase to 1.56 billion by 2025. As at 2015, the burden of hypertension has risen to 1.13 billion with 1 in 4 men and 1 in 5 women being affected [8]. In comparison with other WHO regions, Africa has the highest prevalence of hypertension with an overall prevalence of 46% in adults aged 25 years and above for both sexes combined [9]. Review analysis of the prevalence of hypertension among Nigerian adults showed an estimated prevalence of 28.9% [10] with a range of 6.2–48.9% for men and 10.0–47.3% for women [11] as well as 30.6 and 26.4% among urban and rural dwellers, respectively [10]. In low and middle income countries, hypertension prevalence has been described as increasing [12, 13] mainly due to a rise in hypertension risk factors with many unaware of their status [10, 12, 14].

The fact that most people with hypertension are unaware of their blood pressure status may be responsible for sudden deaths reported orally in the study area. It is not only devastating to affected families but also strengthens cultural misconception that these adults were remotely killed since apparently they were healthy. This calls for assessment of apparently healthy adults for early identification, referral and management of hypertension as this will halt the effects of uncontrolled hypertension which have been described as devastating [15]. There are significant health and economic gains attached to early detection, adequate treatment and good control of hypertension [16] and lack of reliable data has made it very difficult for Nigerian policy-makers to concentrate efforts to control emerging health burden of the disease [17]. Based on this, this study aimed to assess the prevalence and predictors of different patterns of hypertension among apparently healthy adults in three rural communities of Udenu Local Government Area (LGA), Enugu north senatorial zone of Enugu State, Southeast Nigeria.

## Methods

### Study setting

This study was conducted in three rural communities (Obollo-afor, Orba and Ezimo-Ulo) of Udenu Local Government Area, Enugu North senatorial zone of Enugu State, Southeast Nigeria. Udenu LGA has three subunits called development centers (Udenu (North and East), Orba and Udunedem).

### Study design and participants

This study employed a cross-sectional survey design. It included adults (20–60 years) but excluded pregnant women, nursing mothers, sick persons and those with known diagnosis of hypertension or are taking drugs for hypertension.

### Sample size determination

The sample size used for the study was derived through a single population proportion (Cochran’s) formula based on a confidence level of 95 and 5% margin error. Hypertension prevalence of 20% was used; design effect of 2 and 5.0% non-response rate were added to give a total of 517. Probability proportional to size was used to allocate sample sizes to each of the communities thus: Obollo-afor (40%, 207), Ezimo-Ulo (30%, 155) and Orba (30%, 155).

### Sampling technique

A 6-stage sampling technique involving proportionate, systematic and simple random (ballot method) sampling was used in selecting the study participants. Stage one involved selection of a community from each development center using simple random sampling technique (SRST) by balloting while stage two involved selection of two villages from each community by SRST. Stage three involved selection of clans by SRST. Houses in the clans were selected in stage four through systematic random sampling (every 10th house). Sampling interval was obtained by dividing the number of persons to be selected in the clan by the total number of living houses. In houses with more than one household, household selection by SRST were conducted (stage five) and in stage six, only one eligible adult from each selected household was selected through SRST by balloting without replacement.

### Ethical clearance and informed consent to participate

Ethical clearance for the study was obtained from the Health Research Ethics Committee of the University of Nigeria Teaching Hospital (UNTH), Ituku-Ozalla, Enugu State (NHREC/05/01/2008B-FWA00002458-1RB00002323). A detailed explanation of the study protocol was given to the participants after which voluntary informed oral consent to participate was obtained from each of them.

### Data collection methods

Data for this study were collected between June and August, 2018.

#### Questionnaire

An interviewer administered questionnaire was used to obtain data on socio-demographic (age, gender, education and marital status) characteristics of the respondents. Assessment of health status involved requesting respondents to rate their health status as poor, good and excellent. Frequent consumption of fruits, vegetables, nuts and legumes was taken as weekly consumption of 4 to 7 times. To obtain data on alcohol consumption, respondents were asked to recall types and quantity of alcoholic drink normally consumed daily within 12 months preceding data collection. Any consumption above the recommended alcohol intake of 2 drinks per day for males and one drink per day for females was taken as above recommendation irrespective of intake frequency. About 350 ml of beer gives one drink of alcohol.

##### Anthropometry:

Body mass index for each participant was calculated from weight and height measurements obtained through the use of Hanson’s weighing scale (capacity of 120 kg) and a meter rule attached to a wooden pole, respectively. The participants were weighed in light clothing and reading was taken to the nearest 0.1 kg. Height to the nearest 0.1 cm was measured with the participants standing erect on a flat surface. Having a BMI of ≥30 Kg/m^2^ was taken as general obesity. Waist circumference was measured with a flexible non-stretch tape placed on the midpoint between the top of the iliac crest and the bottom of the rib cage where the last palpable rib is found. Values ≥94 cm for males and ≥ 80 cm for females were used to determine the prevalence of abdominal adiposity [18]. The weighing scale was maintained at zero before taking the weight measurements.

#### Clinical examinations

Blood pressure was determined twice (minimum of 3 mins interval was observed) by trained research assistants using Omron automatic sphygmomanometer (M2: HEM-7121-E, Vietnam) with the participant sitting comfortably and arm resting on a table at the same level with the heart. Average of the two readings was used in analysis. The 2017 American College of Cardiology/American Heart Association guideline [1] was used in interpreting the BP: normal SBP as < 120 mmHg and DBP as < 80 mmHg; elevated SBP as 120–129 and DBP as < 80; stage 1 hypertension as SBP of 130–139 and DBP of 80–89 and stage 2 hypertension as SBP of ≥140 and DBP of ≥90. Isolated systolic hypertension (ISH) was taken as SBP ≥130 with DBP < 80, isolated diastolic hypertension (IDH) as DBP ≥80 with SBP < 130 and combined systolic and diastolic hypertension (CSDH) as SBP ≥130 with DBP ≥80. Three different classification standards [1, 18, 19] were used to evaluate overall prevalence of hypertension among the adults: SBP of ≥140 and or DBP of ≥90 [19], SBP of ≥130 and or DBP of ≥85 [18] and SBP of ≥130 and or DBP of ≥80 [1].

#### Quality control

Supervision and technical support were provided to trained research assistants throughout the survey period to ensure study protocols were followed as planned. On the spot random checks of collected data was conducted and identified inconsistencies/missing data were fixed. Weight and blood pressure equipment were checked after each measurement to ensure continued functionality.

#### Statistical analysis

Data collected in the field were entered into Microsoft excel, sorted and cleaned before transfer to SPSS. All statistical analysis were performed with IBM SPSS (IBM Corp., Armonk, New York), version 21.0. Numerical data were presented as means and standard deviations and categorical data as frequencies and percentages. Independent sample T-test, Chi square and analysis of variance were used to evaluate relationships between and among variables. Bivariate and multivariate logistic regression analyses were performed to determine factors in association with ISH, IDH and CSDH, respectively. Crude and adjusted odd ratios with 95% confidence interval and their *p* values were reported for each of the predictor﻿ and outcome variables. Associations with 95% (α = 0.05) precision were accepted as having attained the required level of statistical significance.

## Results

Table 1 shows the general characteristics of the adults. The participants were aged 20–29 (30.6%), 30–39 (19.2%), 40–49 (20.4%) and 50–60 (29.8%) years. Majority (74.8%) was currently married/cohabiting; 8.4% were separated/divorced/widowed. Adults with no formal/primary and secondary education had equal percentage (37.0%). Only 5.4% reported poor health status; more than half (54.8%) did not consume alcohol. Most (83.0%) had abdominal and or general obesity. More respondents consumed legumes (59.2%), and nuts (53.6%) but less consumed fruits (10.2%) and vegetables (35.8%) frequently.

**Table 1:** General characteristics of the respondents. Study on different patterns of hypertension among adults in rural communities of Enugu State, Southeast Nigeria, 2018

Variables	Frequency	Percentage
**Sex**		
Male	213	42.6
Female	287	57.4
**Age (years)**		
20–29	153	30.6
30–39	96	19.2
40–49	102	20.4
50–60	149	29.8
**Marital status**		
Currently married/cohabiting	374	74.8
Never married	84	16.8
Separated/divorced/widowed	42	8.4
**Educational level**		
No formal/primary	185	37.0
Secondary	185	37.0
Tertiary	130	26.0
**Self-perceived health status**		
Poor	27	5.4
Good	358	71.6
Excellent	115	23.0
*Obesity		
Absent	85	17.0
Present	415	83.0
**Alcohol consumption**		
None	274	54.8
Within recommendation	152	30.4
Above recommendation	74	14.8
**Frequent consumption of legumes**		
No	204	40.8
Yes	269	59.2
**Frequent consumption of nuts**		
No	232	46.4
Yes	268	53.6
**Frequent consumption of fruits**		
No	449	89.8
Yes	51	10.2
**Frequent consumption of vegetables**		
No	321	64.2
Yes	179	35.8

Frequent consumption = 4–7 times weekly

*Abdominal and or general obesity

Gender- and age-wise classifications of participants’ blood pressure are presented in Table 2. Male and female mean SBP and DBP were similar (*P* > 0.05) while both SBP and DBP increased significantly with age (*P* < 0.001). Overall prevalence were 10.6% for ISH, 18.2% for IDH and 37.8% for CSDH. Gender-wise difference in the prevalence was strongest for IDH and CSDH (*P* < 0.001). Age-wise differences were significant for ISH (*P* < 0.001), IDH (*P* < 0.01) and CSDH (*P* < 0.001).

**Table 2:** Blood pressure stratified by gender and age. Study on different patterns of hypertension among adults in rural communities of Enugu State, Southeast Nigeria, 2018

Variables	Sex			Age categories in years					All
	Male	Female		20–29	30–39	40–49	50–60		
	**Mean ± SD**		***P*** **value**	**Mean ± SD**				***P*** **value**	**Mean ± SD**
**Mean systolic blood pressure**	130.6 ± 18.300	133.2 ± 21.151	0.142	122.5^a^ ± 12.75	131.3^b^ ± 18.75	137.8^c^ ± 19.29	138.6^cd^ ± 23.39	0.000***	132.1 ± 20.039
**Mean diastolic blood pressure**	80.9 ± 8.335	81.4 ± 12.747	0.616	78.6^a^ ± 8.51	79.2^ab^ ± 11.36	81.8^b^ ± 12.65	84.9^c^ ± 11.28	0.000***	81.2 ± 11.119
**Mean age (years)**	35.6 ± 12.797	41.9 ± 12.291	0.000***						39.3 ± 12.874
	**N(%)**	**N(%)**		**N(%)**	**N(%)**	**N(%)**	**N(%)**		**N(%)**
**Isolated systolic hypertension (ISH)**									
Absent	259 (90.2)	188 (88.3)	0.592	144 (94.1)	83 (86.5)	86 (84.3)	134 (89.9)	0.000***	447 (89.4)
Stage 1 hypertension	14 (4.9)	15 (7.0)		6 (3.9)	9 (9.4)	2 (2.0)	12 (8.1)		29 (5.8)
stage 2 hypertension	14 (4.9)	10 (4.7)		3 (2.0)	4 (4.1)	14 (13.7)	3 (2.0)		24 (4.8)
**Isolated diastolic hypertension (IDH)**									
Absent	250 (87.1)	159 (74.6)	0.000***	112 (73.2)	83 (86.5)	90 (88.2)	124 (83.2)	0.002**	409 (81.8)
Stage 1 hypertension	26 (9.1)	48 (22.6)		38 (24.8)	9 (9.4)	10 (9.8)	17 (11.4)		74 (14.8)
Stage 2 hypertension	11 (3.8)	6 (2.8)		3 (2.0)	4 (4.1)	2 (2.0)	8 (5.4)		17 (3.4)
**Combined systolic and diastolic hypertension (CSDH)**									
Absent	178 (62.0)	133 (62.4)	0.000***	117 (76.5)	61 (65.5)	53 (52.0)	80 (53.7)	0.000***	311 (62.2)
Stage 1 hypertension	9 (3.2)	36 (16.9)		22 (14.4)	3 (3.2)	13 (12.7)	7 (4.7)		45 (9.0)
Stage 2 hypertension	100 (34.8)	44 (20.7)		14 (9.1)	32 (33.3)	36 (35.3)	62 (41.6)		144 (28.8)
**Blood pressure (BP) classification by degree**									
Normal	75 (26.2)	33 (15.5)	0.000***	45 (29.4)	22 (22.9)	13 (12.7)	28 (18.8)	0.000***	108 (21.6)
Elevated BP (prehypertension)	38 (13.2)	21 (9.9)		23 (15.0)	13 (13.5)	12 (11.8)	11 (7.4)		59 (11.8)
Stage 1 hypertension	50 (17.4)	98 (46.0)		65 (42.5)	22 (22.9)	25 (24.5)	36 (24.1)		148 (29.6)
Stage 2 hypertension	124 (43.2)	61 (28.6)		20 (13.1)	39 (40.7)	52 (51.0)	74 (49.7)		185 (37.0)

. **P < 0.01 ***P < 0.001 *P* values in bold were generated through ANOVA. Mean values for age categories with different superscripts in the same row are statistically significant. Other *P* values were generated through Chi square analysis. Classification was done using the 2017 guideline for the prevention, detection, evaluation and management of high blood pressure in adult^1^

**Fig. 1:**
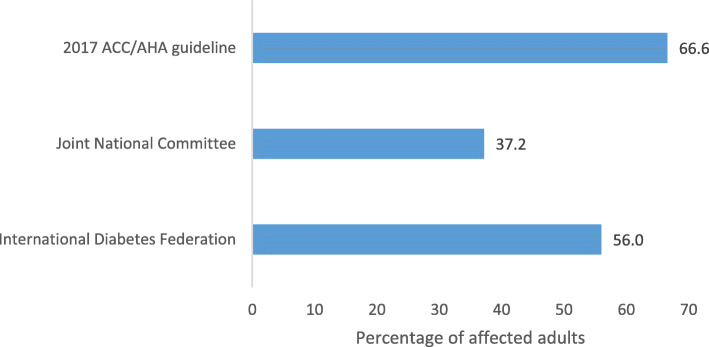
Prevalence of hypertension according to three different standards. Study on different patterns of hypertension among adults in rural communities of Enugu State, Southeast Nigeria, 2018

Prevalence of hypertension among the adults according to three different standards is shown in Fig. 1. The highest prevalence of 66.6% was observed with 2017 ACC/AHA guidelines. Joint National Committee gave the least prevalence of 37.2%. The absolute difference was 29.4% (95% C.I. = 1.60–2.08).

Factors associated with isolated systolic hypertension (ISH) are displayed in Table 3. Age 20–29 (aOR = 0.35, 95% C.I. = 0.13–0.94) and 30–39 (aOR = 0.30, 95% C.I. = 0.11–0.82) years were significantly less likely to have ISH than those aged 50–60 years. Participants with abdominal obesity (aOR = 0.12, 95% C.I. = 0.03–0.56) were at lower risk of ISH than those with combined obesity. Those who perceived themselves as having good (aOR = 3.80, 95% C.I. = 1.29–11.18) and excellent (aOR = 5.28, 95% C.I. = 1.54–18.07) health status were 4 and 5 times at higher risk of ISH, respectively when compared with those who perceived their health status as poor.

**Table 3:** Factors associated with isolated systolic hypertension. Study on different patterns of hypertension among adults in rural communities of Enugu State, Southeast Nigeria, 2018

Variables	Present (***N*** = 53)	Absent (***N*** = 447)	OR(95% C.I.)	aOR(95% C.I.)	***P*** value
**Age (years)**					0.080
20–29	9 (17.0)	144 (32.2)	0.40 (0.16–0.97)*	0.35 (0.13–0.94)*	
30–39	13 (24.5)	83 (18.6)	0.34 (0.14–0.79)*	0.30 (0.11–0.82)*	
40–49	16 (30.2)	86 (19.2)	0.56 (0.24–0.132)	0.49 (0.17–1.46)	
50–60	15 (28.3)	134 (30.0)			
**Sex**					0.600
Male	25 (47.2)	188 (42.1)			
Female	28 (52.8)	259 (57.9)	1.23 (0.70–2.18)	1.22 (0.58–2.58)	
**Education level**					0.338
No formal/primary	25 (47.2)	160 (35.8)	1.77 (0.90–3.48)	1.77 (0.79–3.94)	
Secondary	15 (28.3)	170 (38.0)	1.41 (0.69–2.86)	1.10 (0.48–2.51)	
Tertiary	13 (24.5)	117 (26.2)			
**Obesity**					0.182
None	2 (3.8)	83 (18.6)	0.70 (0.06–7.99)	0.86 (0.07–10.48)	
Abdominal	1 (1.9)	29 (6.5)	0.12 (0.03–0.53)*	0.12 (0.03–0.56)**	
General	26 (49.1)	131 (29.3)	0.21 (0.05–0.89)*	0.35 (0.08–1.63)	
Combined	24 (45.2)	204 (45.6)			
**Self-perceived health status**					0.024*
Poor	7 (13.2)	20 (4.5)			
Good	36 (67.9)	322 (72.0)	3.12 (1.24–7.91)*	3.80 (1.29–11.18)*	
Excellent	10 (18.9)	105 (23.5)	3.68 (1.25–10.80)*	5.28 (1.54–18.07)**	
**Alcohol intake**					0.322
None	28 (52.8)	246 (55.0)			
Within recommendation	14 (26.4)	138 (30.9)	1.12 (0.57–2.20)	1.60 (0.71–3.63)	
Above recommendation	11 (20.8)	63 (14.1)	0.65 (0.31–1.38)	2.04 (0.80–5.20)	
**Frequent fruit consumption**					0.098
No	45 (84.9)	404 (90.4)			
Yes	8 (15.1)	43 (9.6)	0.60 (0.27–1.35)	0.47 (0.19–1.15)	
**Frequent vegetable consumption**					0.148
No	27 (50.9)	294 (65.8)			
Yes	26 (49.1)	153 (34.2)	0.58 (0.33–1.03)	0.63 (0.34–1.18)	
**Frequent nut consumption**					0.130
No	22 (41.5)	210 (47.0)			
Yes	31 (58.5)	237 (53.0)	0.80 (0.45–1.43)	0.61 (0.32–1.16)	
**Frequent legume consumption**					0.286
No	16 (30.2)	188 (42.1)			
Yes	37 (69.8)	259 (57.9)	0.60 (0.32–1.10)	0.69 (0.35–1.36)	

*OR* Odds ratio, *aOR* Adjusted odds ratio *P < 0.05**P < 0.01

Combined obesity, presence of both abdominal and general obesity in an individual

Factors associated with isolated diastolic hypertension (IDH) are revealed in Table 4. Those with secondary education (aOR = 2.05, 95% = 1.02–4.14) were significantly at higher risk of IDH than those with tertiary education. Self-perceived poor health status (aOR = 0.24, 95% C.I. = 0.09–0.65) was associated with lesser likelihood of IDH. Adults without obesity (aOR = 0.10, 95% C.I. = 0.01–0.81) and those with general obesity (aOR = 0.35, 95% C.I. = 0.17–0.72) were less likely to have IDH than those with combined obesity.

**Table 4:** Factors associated with isolated diastolic hypertension. Study on different patterns of hypertension among adults in rural communities of Enugu State, Southeast Nigeria, 2018

Variables	Present (***N*** = 91)	Absent (***N*** = 409)	OR(95% C.I.)	aOR(95% C.I.)	***P*** value
**Age (years)**					0.100
20–29	41 (45.0)	112 (27.4)			
30–39	13 (14.3)	83 (20.3)	0.43 (0.22–0.85)*	0.71 (0.34–1.51)	
40–49	12 (13.2)	90 (22.0)	0.36 (0.18–0.73)**	0.80 (0.36–1.76)	
50–60	25 (27.5)	124 (30.3)	0.55 (0.32–0.96)*	1.82 (0.85–3.87)	
**Sex**					0.229
Male	54 (59.3)	159 (38.9)			
Female	37 (40.7)	250 (61.1)	0.44 (0.27–0.69)***	0.70 (0.40–1.25)	
**Education level**					0.116
No formal/primary	22 (24.1)	163 (39.9)	1.98 (1.12–3.49)*	1.77 (0.90–3.50)	
Secondary	39 (42.9)	146 (35.7)	2.22 (1.22–4.07)*	2.05 (1.02–4.14)*	
Tertiary	30 (33.0)	100 (24.4)			
**Self-perceived health status**					0.001**
Poor	9 (9.9)	18 (4.4)	0.30 (0.13–0.71)**	0.24 (0.09–0.65)**	
Good	47 (51.6)	311 (76.0)	0.88 (0.36–2.14)	0.61 (0.23–1.65)	
Excellent	35 (38.5)	80 (19.6)			
**Obesity**					**0**.012**
None	25 (27.5)	60 (14.7)	0.08 (0.01–0.64)*	0.10 (0.01–0.81)*	
Abdominal	1 (1.0)	29 (6.5)	0.82 (0.46–1.48)	0.56 (0.28–1.09)	
General	40 (44.0)	131 (29.3)	0.30 (0.16–0.55)***	0.35 (0.17–0.72)**	
Combined	25 (27.5)	204 (45.6)			
**Alcohol intake**					0.333
None	48 (52.7)	226 (55.3)	1.41 (0.86–2.30)	1.36 (0.78–2.37)	
Within recommendation	35 (38.5)	117 (28.6)	0.57 (0.26–1.27)	0.74 (0.32–1.71)	
Above recommendation	8 (8.8)	66 (16.1)			
**Frequent fruit consumption**					0.548
Yes	9 (9.9)	42 (10.3)			
No	82 (90.1)	367 (89.7)	0.96 (0.45–2.05)	1.29 (0.57–2.94)	
**Frequent vegetable consumption**					0.955
Yes	31 (34.1)	148 (36.2)			
No	60 (65.9)	261 (63.8)	0.92 (0.57–1.49)	1.02 (0.60–1.71)	
**Frequent nut consumption**					0.853
Yes	49 (53.8)	219 (53.5)			
No	42 (46.2)	190 (46.5)	1.01 (0.64–1.60)	1.05 (0.64–1.72)	
**Frequent legume consumption**					0.858
Yes	53 (58.2)	243 (59.4)			
No	38 (41.8)	166 (40.6)	0.95 (0.60–1.51)	0.95 (0.57–1.58)	

*OR* Odds ratio, *aOR* Adjusted odds ratio *P < 0.05 **P < 0.01 ***P < 0.001

Combined obesity, presence of both abdominal and general obesity in an individual

Table 5 shows the factors associated with combined systolic and diastolic hypertension (CSDH). Secondary (aOR = 0.60, 95% C.I. = 0.36–0.99) and tertiary (aOR = 0.49, 95% C.I. = 0.28–0.85) education were significantly associated with lesser likelihood of CSDH. Having combined obesity (aOR = 4.39, 95% C.I. = 2.25–8.58) placed the participants at 4 times higher risk for CSDH while general obesity (aOR = 1.75, 95% C.I. = 0.87–3.53) almost doubled the risk for CSDH among affected persons though this was not significant.

**Table 5:** Factors associated with combined systolic and diastolic hypertension. Study on different patterns of hypertension among adults in rural communities of Enugu State, Southeast Nigeria, 2018

Variables	Present (***N*** = 189)	Absent (***N*** = 311)	OR(95% C.I.)	aOR(95% C.I.)	***P*** value
**Age (years)**					0.372
20–29	36 (19.0)	117 (37.6)			
30–39	35 (18.5)	61 (19.6)	1.87 (1.07–3.26)*	1.39 (0.74–2.60)	
40–49	49 (25.9)	53 (17.0)	3.01 (1.75–5.15)***	1.77 (0.94–3.34)	
50–60	69 (36.5)	80 (25.8)	2.80 (1.71–4.59)***	1.40 (0.74–2.67)	
**Sex**					0.025*
Male	80 (42.3)	133 (42.8)			
Female	109 (57.7)	178 (57.2)	1.02 (0.71–1.47)	0.57 (0.35–0.93)*	
**Education level**					0.026*
No formal/primary	95 (50.3)	90 (28.9)			
Secondary	57 (30.2)	128 (41.2)	0.42 (0.28–0.65)***	0.60 (0.36–0.99)*	
Tertiary	37 (19.6)	93 (29.9)	0.38 (0.23–0.61)***	0.49 (0.28–0.85)*	
**Self-perceived health statu.bs**					0.253
Poor	9 (4.8)	18 (5.8)			
Good	148 (78.3)	210 (67.5)	1.41 (0.62–3.22)	1.65 (0.66–4.15)	
Excellent	32 (16.9)	83 (26.7)	0.77 (0.31–1.89)	1.11 (0.42–2.99)	
**Obesity**					0.000***
None	15 (7.9)	70 (22.5)			
Abdominal	7 (3.7)	23 (7.4)	1.42 (0.52–3.91)	1.19 (0.41–3.46)	
General	47 (24.9)	110 (35.4)	1.99 (1.04–3.84)*	1.75 (0.87–3.53)	
Combined	120 (63.5)	108 (34.7)	5.18 (2.80–9.59)***	4.39 (2.25–8.58)***	
**Alcohol intake**					0.740
None	94 (49.7)	180 (57.9)			
Within recommendation	61 (32.3)	91 (29.2)	1.28 (0.85–1.93)	1.07 (0.67–1.71)	
Above recommendation	34 (18.0)	40 (12.9)	1.63 (0.97–2.74)	1.25 (0.71–2.19)	
**Frequent fruit consumption**					0.203
Yes	15 (7.9)	36 (11.6)			
No	174 (92.1)	257 (88.4)	0.66 (0.35–1.24)	0.64 (0.32–1.27)	
**Frequent vegetable consumption**					0.344
Yes	64 (33.9)	115 (37.0)			
No	125 (66.1)	196 (63.0)	0.89 (0.61–1.29)	0.82 (0.54–1.24)	
**Frequent nut consumption**					0.893
Yes	101 (53.4)	167 (53.7)			
No	88 (46.6)	190 (46.5)	1.00 (0.70–1.45)	0.97 (0.65–1.45)	
**Frequent legume consumption**					0.942
Yes	112 (59.3)	184 (59.2)			
No	77 (40.7)	127 (40.8)	0.99 (0.69–1.42)	0.99 (0.65–1.48)	

*OR* Odds ratio, *aOR* Adjusted odds ratio *P < 0.05 ***P < 0.001

Combined obesity, presence of both abdominal and general obesity in an individual

## Discussion

This study provides valuable insights into the prevalence, socioeconomic, dietary and lifestyle determinants of three patterns of hypertension within 3 homogeneous rural communities. A response rate of 96.7% was recorded. The study revealed more females than males in line with Enugu State adult population which shows that females outnumber males. Those who were aged 20–39 years were more than other age groups; this agrees with the report of National population commission of Nigeria.

### Prevalence of hypertension and its patterns

The overall prevalence of uncategorized hypertension is higher than previous findings of 28.9% [10] and 33.1% [20]. This high prevalence is a serious cause for worry since the participants were apparently healthy persons unaware of their blood pressure status. Prevalence of ISH reported in this study is comparable to 10.6% [21], higher than 3.4% [22] but lower than 27.6% [23] and more prevalent among females and older participants (≥30–39 years). While the increase with age was not a surprise, the higher female prevalence was contrary to the findings of other researchers [10, 11] and may be a function of age and obesity which affected the females more besides specific risk factors like preeclampsia which contributes to hypertension [24] among them. When compared with hypertensive males who had a 3-fold higher rate than normotensive men, women with hypertension had 6-fold greater rate of coronary heart disease than normotensive women [6].

IDH prevalence with dominance among 20–29 years observed in this study is higher than 10.8% [25] but lower than 19.7% [21] reported earlier. This raises concern as it implies tendency of the blood pressure to increase as the respondents grow older though apparently, they may not be at risk because a previous study reported that IDH was not significantly associated with increased risk for cardiovascular outcomes [26].

The prevalence of CSDH was high in this study when compared to earlier findings of 18.7% [22] and 9.0% [27]. With propensity among males and those aged 40–49 and 50–60 years, it calls for attention because of the danger of organ damage.

The differences in prevalence observed between our findings and those of other researchers may be attributed to the cutoff of ≥140/≥90 mmHg used by these researchers which is higher than the value used in this study.

### Factors associated with ISH, IDH and CSDH

The less likelihood of ISH among 20–29 and 30–39 year-olds suggests increase in prevalence with age. This observation is in line with the report of Ajayi et al. [20] that hypertension was significantly associated with age groups of 30–49 years (OR: 2.258, 95% CI: 1.311–3.884) and ≥ 50 years (OR:7.145, 95% CI:3.644–14.011). This may be attributed to a build-up of risk factors like alcohol consumption, low consumption of fruits and vegetables and the process of aging. With aging, arteries and arterioles become increasingly thickened losing their elasticity and becoming less resilient as blood passes through them.

That ISH was less likely in adults with abdominal obesity and IDH less likely with general obesity than combined obesity suggests how dangerous combined obesity is. It was not a surprise then that those with combined obesity had 4.39 times greater risk of CSDH. This is consistent with current evidence [20, 25, 27, 28] that hypertension was significantly associated with overweight and or obesity. Excess weight gain, especially when associated with increased visceral adiposity is a major cause of hypertension, accounting for 65 to 75% of the risk for primary/essential hypertension [29]. CVDs, the leading cause of mortality worldwide, particularly hypertension and diabetes, are the main illnesses associated with obesity [30]. Obesity and high lipid profile parameters have been shown to be related implying the possibility of atherosclerosis and therefore arteriosclerosis, an important factor in the etiology of hypertension [31]. The high prevalence of obesity observed in this study is worrisome and calls for concerted efforts to create awareness on its consequences and the need for life style modification that will enhance its control.

Self-perceived health status was a significant predictor of ISH and IDH with those who perceived their health status as excellent and good having higher propensity for ISH and IDH than those who perceived theirs as poor. The same pattern was observed with CSDH though it did not reach significant proportion. These participants are unlikely to go for routine health checks and may not adopt control measures since they feel they have no health challenges. The implication is that they are likely to be caught off guard.

Participants with secondary education had 2 times greater likelihood of being affected by IDH than those with tertiary education whereas those with secondary and tertiary education were less likely to have CSDH than those with no formal/primary education. This agrees with the report of other researchers [32–34] showing the role of education in understanding, retention, recall and application of nutrition and health information and knowledge. Educational attainment had a powerful influence on level of blood pressure and may be considered the best predictor of global cardiovascular risk in people with hypertension [33, 34].

Great concern is the percentage of participants with low fruit and vegetable consumption. This agrees with the findings of Kabwama et al. [35] and has implication for higher incidence of non-communicable diseases including cardiovascular diseases of which hypertension is a major risk factor. Adequate consumption of fruits and vegetables is therefore encouraged and as suggested by Borgi et al. [36], greater long-term intake and increased consumption may be advised to reduce the risk of developing (or worsening) hypertension.

### Limitations

The sample size of this study is inadequate for generalization of the research findings to the entire Southeast geopolitical zone of Nigeria. Besides, complex study design was not accounted for in the analysis of data. Cross-sectional design used in this study does not yield data on cause-effect relationships. Some data were self-reported and as such, some degree of bias cannot be ruled out entirely. Our inability to include diabetes, physical activities, tobacco consumption, and dietary intakes of salt, saturated fat and nutrients has created a research gap that can be filled by further researches. This notwithstanding, the study has revealed primary education level, obesity and self-perception of good/excellent health status as modifiable factors associated with hypertension among adults in the study area suggesting intervention programs.

## Conclusion

Prevalence of ISH, IDH and CSDH observed among apparently healthy adults in rural settings was associated with age, obesity, education, self-perceived good and excellent health status. Targeted community-based strategies such as nutrition and health education as well as regular screening for hypertension are required to keep the prevalence under control and avert target organ damage, cerebrovascular accidents and sudden deaths. Addressing issues of education and self-perception of health status has potential to improve understanding of nutrition and health information and encourage life style modification which is aimed at obesity control and therefore hypertension.

## Data Availability

The datasets generated and analyzed in the course of this study are available from the corresponding author on reasonable request.

## Abbreviations

ISH: Isolated systolic hypertension
IDH: Isolated diastolic hypertension
CSDH: Combined systolic and diastolic hypertension
SBP: Systolic blood pressure
DBP: Diastolic blood pressure
LGA: Local Government Area
SRST: Simple random sampling technique
OR: Odds ratio
aOR: Adjusted odds ratio
C.I.: Confidence interval
mmHg: Millimeters of mercury
CVD: Cardiovascular disease

## References

[1] Whelton PK, Carey RM, Aronow WS, Casey DE, Collins KJ, Himmelfarb CD (2018). ACC/AHA 2017 guideline for the prevention, detection, evaluation and management of high blood pressure in adults: A report of the American College of Cardiology/American Heart Association Task Force on Clinical Practice Guidelines. J AM Coll Cardiol.

[2] Bavishi C, Goel S, Messerli FH (2016). Isolated systolic hypertension: an update after SPRINT. Am J Med.

[3] Papademetriou V, Devereux RB, Narayan P, Wachtell K, Bella JN, Gerdts E (2001). Similar effects of isolated systolic and combined hypertension on left ventricular geometry and function: the LIFE study. Am J Hypertens.

[4] Rapsomaniki E, Timmis A, George J, Pujades-Rodriguez M, Shah AD, Denaxas S (2014). Blood pressure and incidence of twelve cardiovascular diseases: lifetime risks, healthy life-years lost, and age-specific associations in 1·25 million people. Lancet.

[5] Rahimi K, MacMahon S (2015). The epidemiology of blood pressure and its worldwide management. Circ Res.

[6] Franklin SS, Wong ND (2013). Hypertension and cardiovascular disease: contributions of the Framingham heart study. Glob Heart.

[7] Kearney PM, Whelton M, Reynolds K, Whelton PK, He J (2005). Global burden of hypertension: Analysis of worldwide data. Lancet.

[8] WHO (2019). Hypertension: Key facts.

[9] WHO (2016). Raised blood pressure: Situation and trends. Global Health Observatory Data.

[10] Adeloye D, Basquill C, Aderemi AV, Thompson JY, Obi FA (2015). An estimate of the prevalence of hypertension in Nigeria a systematic review and meta-analysis. J Hypertens.

[11] Akinlua JT, Meakin R, Umar AM, Freemantle N (2015). Current prevalence pattern of hypertension in Nigeria: A systematic review. PLoS ONE.

[12] Mills KT, Bundy JD, Kelly TN, Reed JE, Kearney PM, Reynolds K, Chen J, He J (2016). Global disparities of hypertension prevalence and control: a systematic analysis of population-based studies from 90 countries. Circulation.

[13] Desormais I, Amidou SA, Houehanou YC, Houinato SD, Gbagouidi GN, Preux PM (2019). The prevalence, awareness, management and control of hypertension in men and women in Benin, West Africa: the TAHES study. BMC Cardiovasc Dis.

[14] Liu X, Hoang VM, Liu Y, Brown RLW. Untreated isolated systolic hypertension among middle-aged and old adults in the United States: Trends in the prevalence by demographic factors during 1999–2010. Int J Chronic Dis. 2015; ID 508584. 10.1155/2015/508584. 10.1155/2015/508584 PMC4590928 26464870

[15] Guwatudde D, Nankya-Mutyoba J, Kalyesubula R, Laurence C, Adebamowo C, Ajayi I, Bajunirwe F, Njelekela M, Chiwanga FS, Reid T, Volmink J, Adami HO, Holmes MD, Dalal S (2015). The burden of hypertension in sub-Saharan Africa: a four-country cross sectional study. BMC Pub Health.

[16] World Health Organization (2013). A global brief on hypertension: Silent killer, global public health crises (World Health Day 2013).

[17] Akinroye K (2013). Nigerians wake up to high blood pressure. Bull World Health Organ.

[18] Chobanian AV, Bakris GL, Black HR, Cushman WC, Green LA, IzzoJr JL (2003). And the national high blood pressure education programme coordinating committee. The seventh report of the joint National Committee on prevention, detection, evaluation, and treatment of high blood pressure: the JNC 7 report. JAMA.

[19] The IDF Consensus worldwide definition of the metabolic syndrome. Belgium: international diabetes federation; 2006. International Diabetes Federation. https://www.idf.org/e-library/consensus-statements/60-idfconsensus-worldwide-definitionof-the-metabolic-syndrome.html.

[20] Singh S, Shankar R, Singh GP. Prevalence and associated risk factors of hypertension: a cross-sectional study in urban Varanasi. Int J Hypertens. 2017; ID 5491838. 10.1155/2017/5491838. 10.1155/2017/5491838 PMC5733954 29348933

[21] Li L, Deng Q (2018). Prevalence of isolated systolic hypertension in middle-aged Chinese. J Hypertens.

[22] Dagnew B, Yeshaw Y (2019). Predictors of isolated systolic hypertension among type 2 diabetes mellitus patients in Jimma University Specialized Hospital, Southwest Ethiopia. BMC Res Notes.

[23] Tan YY, Gast GC, van der Schouw YT (2010). Gender differences in risk factors for coronary heart disease. Maturitas.

[24] Liu F, Adi D, Xie X, Li X-M, Fu Z-Y, Shan CF, Huang Y, Chen BD, Gai MT, Gao XM, Ma YT, Yang YN (2015). Prevalence of isolated diastolic hypertension and associated risk factors among different ethnicity groups in Xinjiang, China. PLoS ONE.

[25] McEvoy JW, Daya N, Rahman F, Hoogeveen RC, Blumenthal RS, Shah AM (2020). Association of isolated diastolic hypertension as defined by the 2017 ACC/AHA blood pressure guideline with incident cardiovascular outcomes. JAMA.

[26] Ajayi IO, Sowemimo IO, Akpa OM, Ossai NE (2016). Prevalence of hypertension and associated factors among residents of Ibadan north local government area of Nigeria. Nig J Cardiol.

[27] Saeed AAW (2017). Combined systolic diastolic hypertension among adults in Saudi Arabia: prevalence, risk factors and predictors: Results of a national survey. Int J Med Res Health Sci.

[28] Cuschieri S, Vassallo J, Calleja N, Pace N, Mamo J. The effects of socioeconomic determinants on hypertension in a cardiometabolic at-risk European country. Int J Hypertens. 2017; ID 7107385. 10.1155/2017/7107385. 10.1155/2017/7107385 PMC5592416 28932598

[29] Hall JE, do Carmo JM, da Silva AA, Wang Z, Hall ME (2015). Obesity-induced hypertension, interaction of neurohumoral and renal mechanisms. Circ Res.

[30] Jiang S, Lu W, Zong X, Ruan H, Liu Y (2016). Obesity and hypertension (review). Exp Ther Med.

[31] Michael OA, Bimbola FM, Rotimi O (2019). The relationship between measures of obesity and atherogenic lipids among Nigerians with hypertension. Mal Med J.

[32] Liu X, Rodriguez CJ, Wang K (2015). Prevalence and trends of isolated systolic hypertension among untreated adults in the United States. J Am Soc Hypertens.

[33] Wang Y, Chen J, Wang K, Edwards CL (2006). Education as an important risk factor for the prevalence of hypertension and elevated blood pressure in Chinese men and women. J Hum Hypertens.

[34] Chiara TD, Scaglione A, Corrao S, Argano C, Pinto A, Scaglione R (2017). Education and hypertension: impact on global cardiovascular risk. Acta Cardiol.

[35] Kabwama SN, Bahendeka SK, Wesonga R, Mutungi G, Guwatudde D (2019). Low consumption of fruits and vegetables among adults in Uganda: findings from a countrywide cross-sectional survey. Arch Public Health.

[36] Borgi L, Muraki I, Satija A, Willett WC, Rimm EB, Foreman JP (2016). Fruit and vegetable consumption and the incidence of hypertension in three prospective cohort studies. Hypertens.

